# Correction of severe valgus osteoarthritis by total knee arthroplasty is associated with increased postoperative ankle symptoms

**DOI:** 10.1007/s00167-020-06246-4

**Published:** 2020-08-24

**Authors:** Frank Graef, Hagen Hommel, Roman Falk, Serafeim Tsitsilonis, Robert Karl Zahn, Carsten Perka

**Affiliations:** 1 Center for Musculoskeletal Surgery, Charité-University Medicine Berlin, Charitéplatz 1, 10117 Berlin, Germany; 2 Department of Orthopedics, Märkisch-Oderland Hospital, Brandenburg Medical School Theodor Fontane, Wriezen, Germany

**Keywords:** Total knee arthroplasty, Total knee replacement, Ankle joint, Osteoarthritis knee, Interplay knee ankle

## Abstract

**Purpose:**

The aim of this study was to assess the mid-term clinical outcome of the ankle joint after total knee arthroplasty (TKA) in high-grade valgus osteoarthritis.

**Methods:**

In this case–control study, *n* = 36 patients with a preoperative mechanical tibiofemoral angle (mTFA) ≥ 15° who underwent TKA between December 2002 and December 2012 were included. The control group (mTFA < 15°) of *n* = 60 patients was created using case matching. Radiological [mechanical tibiofemoral angle (mTFA) and ankle joint orientation to the ground (G-AJLO)] and clinical parameters [Foot Function Index (FFI), Knee Society Score, Forgotten Joint Score, and Range of Motion (ROM)] were analysed. The mean follow-up time was 59 months (IQR [56, 62]).

**Results:**

The degree of correcting the mTFA by TKA significantly correlated with the postoperative FFI (*R* = 0.95, *p* < 0.05), although the knee and ankle joint lines were corrected to neutral orientations. A cut-off value of 16.5° [AUC 0.912 (0.85–0.975 95% CI), sensitivity = 0.8, specificity = 0.895] was calculated, above which the odds ratio (OR) for developing ankle symptoms increased vastly [OR 34.0 (9.10–127.02 95% CI)]. ROM restrictions of the subtalar joint displayed a strong significant correlation with the FFI (*R* = 0.74, *p* < 0.05), demonstrating that decreased ROM of the subtalar joint was associated with aggravated outcomes of the ankle joint.

**Conclusions:**

In this study, higher degrees of leg axis correction in TKA were associated with increased postoperative ankle symptoms. When TKA is performed in excessive valgus knee osteoarthritis, surgeons should be aware that this might trigger the onset or progression of ankle symptoms, particularly in cases of a stiff subtalar joint.

**Level of evidence:**

III.

## Introduction

An increasing specialisation in total knee arthroplasty (TKA) has led to satisfactory patient-related outcomes after TKA, with increasing survivorship rates of the prostheses [11]. Albeit the merits of specialisation are undeniable, it is obvious that joints are tightly connected to each other on a functional level and changes made to a single joint can have a crucial impact on neighbouring joints, as well [1, 18, 32]. For instance, a case was reported in which isolated ankle symptoms were successfully treated by high tibial osteotomy at the knee joint [4].

Clinical experience has shown that patients who presented with excessive valgus osteoarthritis of the knee and underwent TKA frequently complained about ankle symptoms after the operation. A search of the relevant literature on this subject resulted in scattered reports only [7, 30]. Until now, research on the interplay between both joints was primarily centred on radiological changes, but valid clinical data are still missing [12, 15, 22, 31]. Therefore, the primary aim of this study was to assess the mid-term clinical outcome of the ankle joint after TKA in high-grade valgus osteoarthritis. The hypothesis of this study was that the degree of correction of the mechanical tibiofemoral angle (mTFA) by TKA in valgus osteoarthritis correlated with worse outcomes in the ankle joint. Subsequently, the null hypothesis was that there was no correlation between mTFA correction and postoperative ankle symptoms.

## Methods

In this case–control study, patients who presented with preoperative valgus osteoarthritis of the knee and underwent TKA were retrospectively analysed. All operations were performed at a single board-certified joint replacement centre between December 2002 and December 2012. Patients were subdivided into four groups according to their preoperative mTFA, as previously reported [5]: (1) 0 to less than 5°, (2) 5 to less than 10°, (3) 10 to less than 15°, and (4) ≥ 15°. At first, all patients with a valgus mTFA ≥ 15° (*n* = 91 patients) were contacted. Of those 91 patients, 24 could not be contacted, 11 declined to participate in this study, 12 had already died, and 8 could not be included due to compliance (e.g., dementia), leaving *n* = 36 patients with a preoperative valgus mTFA of ≥ 15° for inclusion (Fig. 1). Afterwards, the characteristics of these 36 patients were matched according to their age and body mass index (BMI) to those patients in groups 1–3 (valgus < 15°). Patients in group 1–3 were contacted for participation in this study in order of their closest matching. The number of patients drawn from each group was approximately chosen according to previously published mTFA distribution histograms of patients with valgus osteoarthritis of the knee [17]. Subsequently, for each of the groups 1–3, 20 patients with the closest matching of age, American Society of Anesthesiologists (ASA), and BMI were selected. The median follow-up time was 59 months (IQR [56, 62]). A total of *n* = 96 patients (0° to ≤ 5°, *n* = 20; 5° to ≤ 10°, *n* = 20; 10° to ≤ 15°, *n* = 20; and ≥ 15°, *n* = 36) with a median follow-up time of 59 months (IQR [56, 62]) were included in this study.

**Fig. 1:**
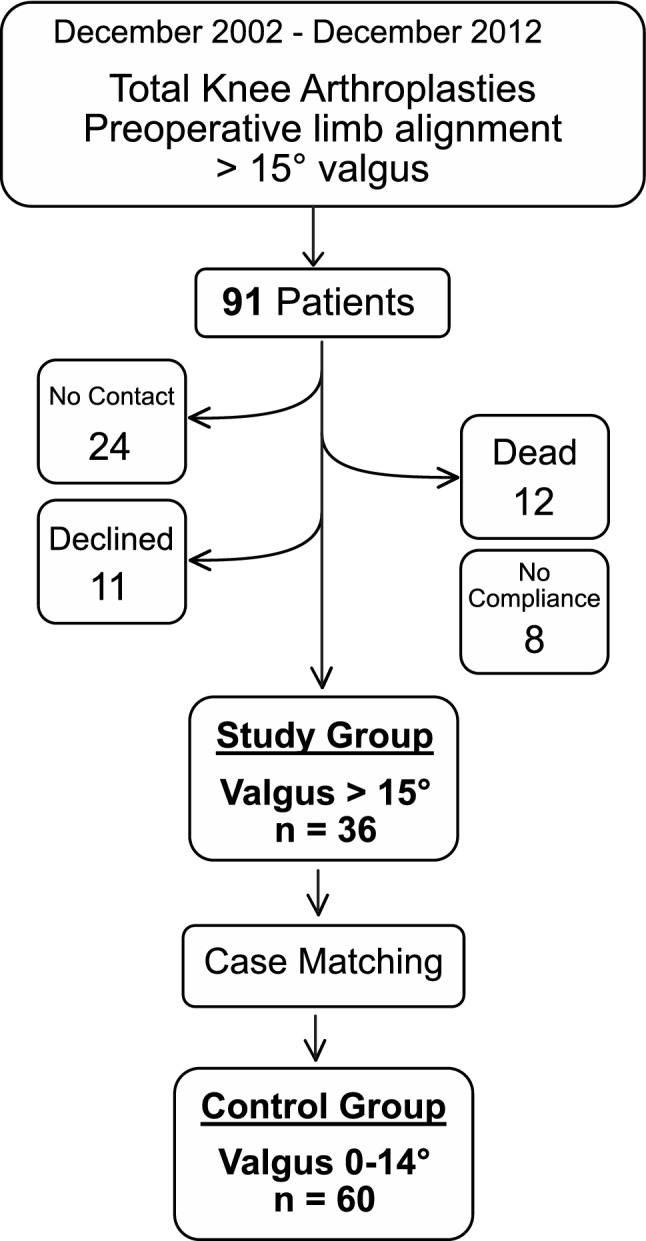
Flowchart of the patient selection process. Between December 2002 and December 2012, a total of 91 patients with a preoperative valgus alignment > 15° underwent TKA. Of these, 24 patients could not be contacted, 12 had died, 11 declined to participate, and 8 could not be included in the study due to lack of compliance (e.g., dementia). Therefore, 36 patients with a preoperative valgus alignment > 15° were included in the study. Patients in the categories 0°–4°, 5°–9°, and 10°–14° preoperative valgus alignment were manually matched according to age, ASA, and BMI of the cohort > 15°

All TKAs included in this study were performed using the Journey II BCS system (Smith & Nephew, London, UK) and the RT-PLUS system (Smith & Nephew, London, UK) or its modular version if a wedge was necessary. Alignment correction was achieved using a measured resection technique with ligament balancing, if necessary [32].

Two time points were analysed in this study: 1 week before the operation (radiological analysis) and at the follow-up visit (minimum follow-up time = 4 years) (radiological and clinical analyses). A single observer (HH) measured specific angles in the radiographs taken 1 week before the operation and at the follow-up visit, including the mTFA and G-AJLO. In addition, the mechanical lateral distal femur angle (mLDFA) and lateral distal tibia angle (LDTA) were measured to account for changes and differences of knee and ankle phenotypes [8, 9, 19]. The mTFA was measured, as previously described, as the angle between the femoral and tibial mechanical angle [23]. The G-AJLO was measured, as described by Lee et al., as the angle between the tangent to the subchondral plate of the talus and the horizontal grid line on radiographs [15]. Varus angles were noted as positive values, and valgus angles were noted as negative values.

At the follow-up visit, patients were asked to state whether postoperative ankle pain was higher than before the TKA (yes/no). According to these results, all patients were subdivided into two groups: patients without (group A) and with (group B) increased postoperative ankle pain. Group A consisted of *n* = 76 patients. Group B consisted of *n* = 20 patients. Patient baseline characteristics are displayed in Table 1. They were furthermore asked to state whether postoperative knee pain was higher (yes/no) and to quantify pain at the knee and ankle joint using the numerical rating scale (0–10; 0 = no pain; 10 = worst imaginable pain). Further assessed factors included weight, age, and BMI at the time of the follow-up visit; postoperative complications (e.g., thrombosis and prolonged wound healing); hindfoot deformities (pes planovalgus/pes cavovarus); and injuries of the ankle or foot before or after TKA. All patients participating in this study were clinically examined for the maximum flexion of the knee joint, as well as the ROM of the ankle (at 90° knee flexion) and the subtalar joint (measured as eversion and inversion of the calcaneus in prone position). ROM was categorised as follows: 0 = no limitation; 1 = 0°–5° limitation; 2 = 6°–10° limitation; 3 =  ≥ 10° limitation; and 4 = ankylosis. The grade of limitations referred to extension and flexion in the knee and ankle joints, and to eversion and inversion in the subtalar joint.

**Table 1:** Baseline characteristics of our study cohort with preoperative valgus osteoarthritis of the knee

Increased postoperative ankle pain	No (group A)	Yes (group B)	*p*
*n* =	76	20	
Age	67.3 (3)	68.8 (2)	0.346
Sex = male (%)	28 (36.8)	2 (10.0)	0.029
BMI	30.4 (18)	30.1 (14.6)	0.775
ASA	1.9 (2)	2.1 (2)	0.154
System			< 0.001
Journey II BCS	56 (73.7)	4 (20.0)	
RT-PLUS (modular)	20 (26.3)	16 (80.0)	
Postoperative complications = *n* (%)	4 (5.3)	2 (10)	0.225

Values are given as median with interquartile ranges (square brackets) (non-normal distribution) or as means with ranges (normal distribution). Significance level *p* < 0.05. Two-sided *t* tests applied for normal distribution, two-sided Mann–Whitney *U* test for non-normal distribution, and Fisher’s exact test for categorial variables

The clinical outcome of the knee joint was evaluated using the Knee Score (KS), the Function Score (FS), and the Knee Society Score (KSS), as well as the Forgotten Joint Score (FJS). The Foot Function Index (FFI) was used to evaluate the outcome of the ankle joint. The FFI is a two-part score including a pain (FFI Pain) and function (FFI Function) scale with higher points correlating to worse outcomes. The FFI is reported for each scale separately and as the sum of both scales (FFI sum) [21]. All scores were assessed using cross-cultural adaptations of the questionnaires in the German language [2, 10, 21]. In the KS, FS and FJS higher points correspond to better outcomes.

Data were analysed for normal/non-normal distribution using histograms, QQ plots, means/medians, and skewness. Correlations were displayed with scatter plots and calculated using Pearson’s correlation coefficient, and cut-off values were determined using receiver-operating characteristic (ROC) curves for maximum sensitivity and specificity. Differences between two independent groups with normal distribution were calculated using two-sided *t* tests and with non-normal distribution using two-sided Mann–Whitney *U* tests. The exact Fisher’s test was used for independent categorical variables. The significance level was set for *p* < 0.05. Bonferroni correction was applied for multiple comparisons. All statistical calculations and figures were performed using “R” and the software RStudio© (RStudio, Inc., Boston, USA). Post hoc power analysis was done using G*Power (HHU Düsseldorf, Germany), which demonstrated a power of 92% for a sample size of *n* = 96 and an alpha error of 0.05 with a medium effect size of *d* = 0.3.

This study was approved by the local ethics committee (AS17(bB)/2015). The written informed consent of all patients was obtained.

## Results

The FFI was significantly higher in group B than in group A (Table 2). The degree of mTFA correction and the preoperative mTFA were significantly higher in group B compared to group A (Table 3). Scatter plots and correlation analysis revealed a strong positive correlation between the postoperative FFI sum score and the degree of operative mTFA correction (*R* = 0.95, *p* < 0.001) (Fig. 2). Subsequently, the calculation of a cut-off value regarding the degree of correction and its impact on increased postoperative ankle pain was undertaken. The ROC curve rendered a cut-off value of 16.5° for valgus osteoarthritis of the knee [AUC 0.912 (0.85–0.975 95% CI), sensitivity = 0.8, and specificity = 0.895] (Figs. 3, 4). The odds ratio (OR) for developing increased postoperative ankle pain when correction was performed beyond this cut-off value was OR = 34.0 (9.10–127.02 95% CI).

**Table 2:** Clinically assessed parameters

Increased postoperative ankle pain	No (group A)	Yes (group B)	*p*
Foot Function Index—pain	18.0 [15.8, 27.3]	51.0 [34.8, 67.3]	< 0.001
Foot Function Index—function	19.5 [17.0, 31.0]	56.5 [48.0, 63.3]	< 0.001
Foot Function Index—sum	37.5 [32.8, 60.0]	109.0 [88.3, 127.0]	< 0.001
Knee Score	88.0 [85.0, 90.0]	82.0 [80.0, 84.0]	< 0.001
Function Score	87.5 [84.0, 90.0]	77.0 [73.0, 82.5]	< 0.001
Knee Society Score	175.5 [170.0, 179.3]	157.5 [154.0, 165.3]	< 0.001
Forgotten Joint Score (TKA)	48.0 [42.0, 65.8]	36.0 [33.0, 38.0]	< 0.001
Maximum flexion knee joint (°)	120.0 [110.0, 125.0]	115.0 [110.0, 120.0]	0.054
Foot deformity = *n* (%)	12 (15.8)	6 (25)	0.459
Preoperative foot/ankle trauma (%)	8 (10.5)	1 (5.0)	0.680
Postoperative foot/ankle trauma (%)	8 (10.5)	2 (10.0)	1.000
ROM ankle joint	1.0 [0.0, 2.0]	3.0 [2.0, 3.0]	< 0.001
ROM subtalar joint	1.0 [1.0, 2.0]	3.0 [2.0, 3.0]	< 0.001
Pain knee—numerical rating scale	2.0 [2.0, 3.0]	3.0 [2.0, 3.0]	0.067
Pain ankle—numerical rating scale	1.0 [0.0, 2.0]	4.0 [4.0, 5.0]	< 0.001

Unless stated otherwise, the parameters were assessed postoperatively. Values are given as median with interquartile ranges (square brackets) (non-normal distribution) or as means with ranges (normal distribution). Significance level *p* < 0.05. Two-sided *t* tests applied for normal distribution, two-sided Mann–Whitney *U* test for non-normal distribution, and Fisher’s exact test for categorical variables

**Table 3:** Radiological measurements subdivided by the time point at which they were assessed (preoperative and postoperative) as well as the comparison of each from both time points

Increased postoperative ankle pain	No (group A)	Yes (group B)	*p*
Preoperative			
mTFA (°)	− 9.0 [− 4.0, − 15.0]	− 21.0 [− 18.0, − 24.5]	< 0.001
G-AJLO (°)	− 5.0 [− 3.0, − 9.0]	− 16.0 [− 11.3, − 17.0]	< 0.001
mLDFA (°)	82.0 [77.0, 84.0]	72.5 [70.0, 75.5]	< 0.001
MPTA (°)	90.0 [89.0, 92.0]	93.5 [92.0, 95.3]	< 0.001
LDTA (°)	90.0 [89.0, 91.3]	92.5 [92.0, 94.0]	< 0.001
Postoperative			
mTFA (°)	− 2.0 [0.0, − 4.0]	0.0 [2.0, − 1.0]	0.001
G-AJLO (°)	− 2.0 [− 1.0, − 2.0]	− 2.0 [− 2.0, − 3.0]	0.001
mLDFA (°)	87.0 [86.0, 89.0]	88.5 [88.0, 90.0]	0.016
MPTA (°)	90.0 [89.0, 90.0]	90.0 [90.0, 91.0]	0.011
LDTA (°)	90.0 [88.8, 91.0]	92.0 [91.8, 93.0]	< 0.001
Comparison pre- to postoperative			
Difference pre- and postop mTFA (°)	5.0 [2.0, 14.0]	20.0 [17.0, 23.0]	< 0.001
Difference pre- and postop G-AJLO (°)	4.0 [2.0, 7.0]	14.0 [7.8, 15.0]	< 0.001
Difference pre- and postop mLDFA (°)	4.0 [2.8, 10.3]	17.0 [12.8, 18.8]	< 0.001
Difference pre- and postop MPTA (°)	1.0 [0.0, 2.0]	3.0 [2.0, 5.0]	0.001
Difference pre- and postop LDTA (°)	0.0 [0.0, 1.0]	0.0 [0.0, 2.0]	0.031

Values are given as median with interquartile ranges (square brackets) (non-normal distribution) or as means with ranges (normal distribution). Significance level *p* < 0.05. Two-sided *t* tests applied for normal distribution, two-sided Mann–Whitney *U* test for non-normal distribution, and Fisher’s exact test for categorical variables

*mTFA* mechanical tibiofemoral angle, *G-AJLO* ankle joint line orientation to the ground, *mLDFA* mechanical lateral distal femur angle, *LDTA* lateral distal tibia angle

**Fig. 2:**
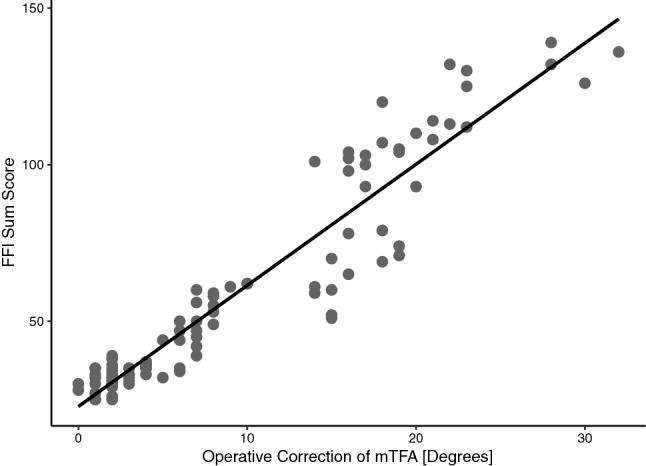
Scatter plot and linear regression line. Each point represents one patient. Pearson’s correlation analysis showed a strong significant correlation between the degree of operative correction of the mechanical tibiofemoral angle (mTFA) and the Foot Function Index score (*R* = 0.95, *p* < 0.001). This indicates that higher degrees of mTFA corrections in valgus osteoarthritis of the knee are associated with worse outcomes of the ankle joint

**Fig. 3:**
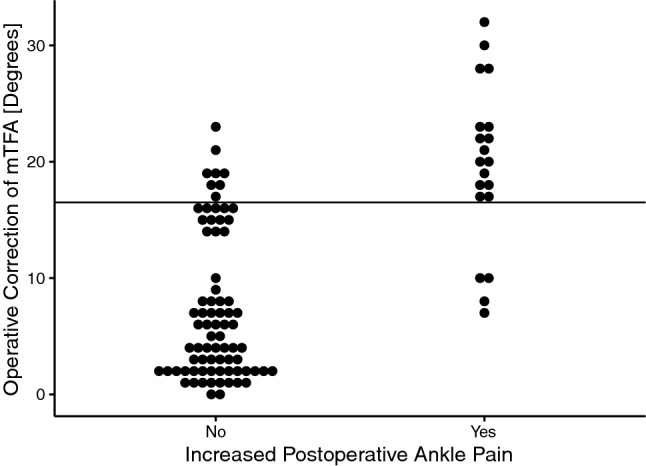
Dot plot displaying the distribution of patients with (yes) and without (no) increased ankle pain after total knee arthroplasty (TKA) on the *x*-coordinate depending on the degree of operative correction of the mechanical tibiofemoral angle (*y*-coordinate). Each point represents one patient. The horizontal line displays the cut-off value of 16.5°, above which the odds ratio for developing ankle pain after TKA increased vastly [OR = 34.0 (9.10–127.02 95% CI)]

**Fig. 4:**
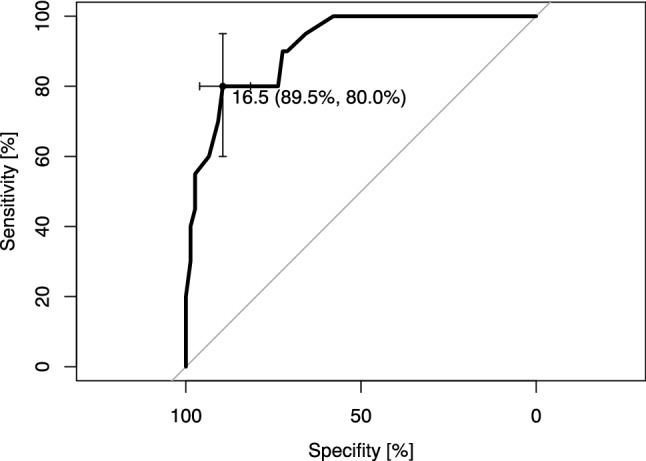
Receiver-operating characteristic curve. The calculation for maximum sensitivity and specificity rendered a cut-off value of 16.5° concerning the operative correction of the mechanical tibiofemoral angle [AUC 0.912 (0.85–0.975 95% CI), sensitivity = 0.8, and specificity = 0.895]. If correction was performed beyond this cut-off, the odds ratio for developing ankle pain after TKA increased manifold [OR = 34.0 (9.10–127.02 95% CI)]

The ROM of the ankle and subtalar joints were significantly reduced in group B compared to group A (Table 2). Lower ROM of the subtalar joint significantly correlated with higher FFI sum scores (*R* = 0.74, *p* < 0.001). Lower ROM of the subtalar joint also significantly correlated with the degree of operative mTFA correction (*R* = 0.76, *p* < 0.001). Further characteristics such as foot deformities (pes planovalgus/pes cavovarus) or pre-/postoperative ankle trauma were not significantly different between both groups (Table 2). Baseline characteristics are reported in Table 1.

Correlation analysis displayed a strong positive correlation between the preoperative G-AJLO and the preoperative mTFA (*R* = 0.94, *p* < 0.001). Before TKA, the G-AJLO was significantly reduced to values of horizontal alignment in both groups (Table 3). Postoperatively, there was no significant correlation between the G-AJLO and mTFA (*R* = − 0.12, *p* = 0.21). Over the entire study cohort from preoperative to postoperative, the mLDFA significantly increased (preoperative 80° [IQR 71.0, 89.0], postoperative 88° [IQR 76.0, 84.0], *p* < 0.001), the G-AJLO significantly decreased (preoperative 8° [IQR − 0.5, 16.5], postoperative 2° [IQR 1.0, 3.0], *p* < 0.001), and the LDTA did not change significantly (preoperative 91.0° [IQR 88.0, 94.0], postoperative 90° [IQR 89.0, 92.0], *p* > 0.44). Table 3 gives an overview of radiological changes from both groups before and after TKA.

It could be demonstrated that the operative correction of the mTFA had a significant negative correlation with the KSS (*R* = − 0.77, *p* < 0.001) and FJS (*R* = − 0.73, *p* < 0.001). The results from postoperative knee and ankle scores are reported in Table 2.

## Discussion

The most important finding of this study was that the degree of coronal knee axis correction by TKA was associated with increased pain and deteriorated function of the ankle joint, although coronal ankle joint orientations were reinstituted to a neutral alignment. TKA used for the treatment of valgus knee osteoarthritis had a crucial impact on the alignment and clinical outcome of the ankle joint.

This study is the first study to analyse the influence of TKA in valgus osteoarthritis of the knee on the ankle joint. The previous studies have already shown that TKA affected the ankle joint in varus knee osteoarthritis [5, 6, 12]. Gursu et al. retrospectively analysed the influence of TKA on the ankle joint with a cohort of 80 patients, who all had a preoperative varus deformity > 10° [6]. Nineteen patients complained about increased ankle pain after TKA. Thirteen of these 19 patients had a preoperative mTFA > 15°, corresponding to the cut-off values previously reported for varus osteoarthritis [5]. Kim et al. performed a prospective study on 55 patients, who presented with varus osteoarthritis of the knee and underwent TKA. In 12.3% of the cases, patients experienced aggravated ankle symptoms after the operation [12]. In a recently published study, it could be demonstrated that the degree of knee axis correction by TKA in varus osteoarthritis of the knee significantly correlated with increased pain and decreased function of the ankle joint. The authors could, furthermore, show that the aggravated ankle symptoms were associated with decreased ROM of the subtalar joint, delivering a first possible explanation for the increased pain [5]. In our study, decreased ROM of the subtalar joint was associated with aggravated outcomes of the ankle joint, too.

Radiological studies about the relationship between the knee and ankle axis likewise have emphasised the importance of the subtalar joint [16, 22, 28, 29]. Radiological parameters of the ankle joints of patients who underwent TKA for varus knee osteoarthritis were analysed by Lee et al. It was found that, in cases of a stiff subtalar joint, compensation of knee varus might take place in the ankle joint, ultimately leading to osteoarthritis [14]. Biomechanical cadaver studies and radiological surveys have confirmed these observations [13, 14, 26]. In valgus osteoarthritis of the knee, after TKA, ankle symptoms may depend on the eversion capability of the subtalar joint given that the knee valgus was compensated by the subtalar joint remaining in inversion position for a long time. A more in-depth analysis is required in future clinical studies to analyse if the subtalar joint is fixed in an eversion or inversion position and to demonstrate the exact ROMs thereof. Moreover, it remains to be evaluated whether conservative measures, such as manual therapy or orthopaedic insoles, are effective in treating ankle symptoms after TKA, which are induced by a fixed subtalar joint.

In this study, patients who did not complain about increased ankle pain after TKA remained in slight valgus after the operation, whereas patients with increased ankle pain after TKA had been corrected to a neutral axis (Table 3). In contrast, Kim et al. found that patients with varus osteoarthritis of the knee who underwent TKA and had increased postoperative ankle pain were left in slight varus compared to patients who did not complain about ankle pain [12]. The classical concept in TKA to correct each patient’s mechanical lower leg axis to a neutral alignment (0°) is currently being discussed, because, for some patients, the natural lower leg axis differs from 0° [8, 19, 24]. Therefore, an overcorrection or undercorrection of the lower leg axis by TKA may also be responsible for increased ankle symptoms. It remains to be analysed whether kinematically aligned TKA, in which forcing the patient into a neutral axis is not the primary goal, does lead to fewer patients with ankle problems after surgery.

In the present study, TKA corrected the valgus malalignment for both the knee and ankle joints. The anatomical geometry of the ankle joint, however, was not altered, as is the case in high tibial osteotomies [3]. Therefore, it seems surprising that ankle symptoms manifested and increased, nonetheless (Fig. 5). One possible explanation for this phenomenon could be that the ankle symptoms had already been present before the operation, but had been masked by the pain in the knee joint and limited physical activity [27]. On the other hand, a radiological study could demonstrate that patients developed radiological signs of ankle osteoarthritis only after TKA [14]. Nevertheless, little is known about the complex interplay between the knee and ankle joints, particularly in regard to changes of the ligaments and tendons of the ankle after TKA. In the knee joint, restoring a neutral axis in excessive valgus or varus osteoarthritis renders a ligamentous mismatch between the medial and lateral collateral ligaments. This mismatch is addressed accordingly during the operative procedure [20]. It remains to be investigated if the same imbalance is being caused in the ankle joint by the prompt changes made to the mechanical axis through TKA. In excessive valgus knee osteoarthritis, fixed flexion deformities may force the ankle joint to go into dorsiflexion, which could have caused tibiotalar impingement in the ankle joint.

**Fig. 5:**
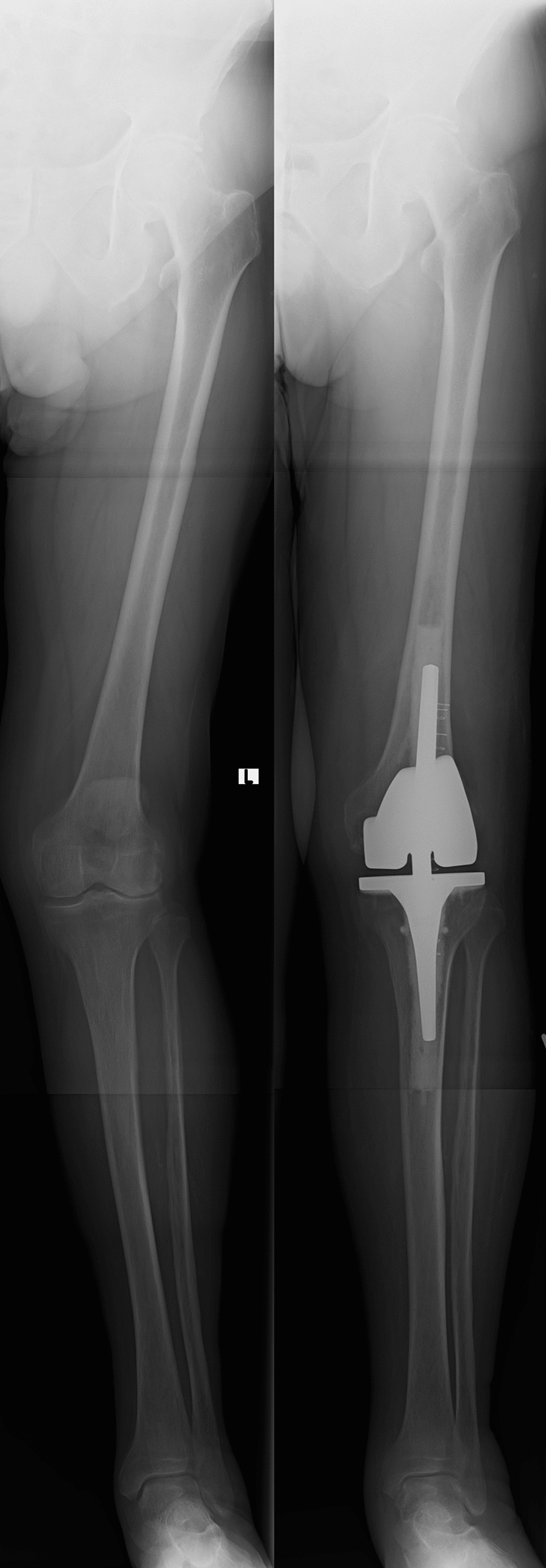
Preoperative (left) and postoperative (right) X-ray of 72-year-old male patient who presented with 16° valgus knee osteoarthritis, which was corrected to a neutral alignment through TKA. Although the G-AJLO was corrected from 10° valgus to 4°, the patient developed ankle pain after the operation (follow-up time 48 months). The LDTA did not change from the preoperative to postoperative period (93°)

The main limitation of the present study was the selection bias. Of 91 patients with a preoperative mTFA ≥ 15°, 55 patients were excluded. To tackle this selection bias, the patients in the control group (mTFA < 15°) were matched for age, ASA, and BMI to generate homogenous baseline characteristics. Although case matching was performed to create a control group, selection bias could have been rendered in the control group given the small sample size, too. Another limitation was the retrospective study design. Therefore, for example, preoperative clinical scores could not be analysed, and specific parameters of the ankle joint, such as the grade of osteoarthritis, fixed flexion deformities, or talar tilt, could not be assessed [25]. Since the preoperative FFI was not available in this study, patients were asked after surgery if they had experienced increased ankle pain, in an effort to compare the clinical state of the joint from the preoperative to postoperative period. Fixed flexion deformities, for instance, may have rendered our radiological measurements on the whole-leg radiographs imprecise [33]. Furthermore, sagittal changes of the knee and ankle joint, such as tibiotalar impingement signs, were not measured and should be taken into account for future studies. A further limitation of this study is that, in groups A and B, different prosthesis designs, which have different functions, were implanted. Since patients in group B had a significantly higher mTFA, these patients often required a hinged prosthesis design in case of ligamentous instability. Patients in group A had a significantly lower mTFA and were, therefore, primarily implanted with non-hinged prostheses in case of ligamentous stability. The choice of TKA implants could have had an effect on the ankle function. Because of ethical issues, choosing the same implants for all patients regardless of their mechanical axes was not possible, but the impact of the prosthesis design on the ankle joint should be discussed, nevertheless.

The results of this study have some major clinical implications. First, before performing TKA, the geometry of the knee and ankle joints should be analysed together. This would enable the surgeon to both detect preoperative malalignments of the ankle joint and simulate how the geometry and joint line of the ankle would alter if TKA was performed. Second, before performing TKA, the ankle and subtalar joints should routinely be clinically examined to detect preoperative pathologies, such as decreased dorsiflexion in cases of tibiotalar impingement or a stiff subtalar joint, which would not be able to compensate for changes made to the mechanical leg axis. In future clinical trials, assessing more comprehensive clinical data preoperatively could help to explain the mechanisms that led to the increased ankle pain.

## Conclusion

This study demonstrated for the first time that the extent of correcting the mechanical leg axis alignment in valgus osteoarthritis by TKA is associated with postoperative ankle symptoms.
